# Application of a Laser Profile Sensor for the Full-Field Measurement of the Continuous Icing Process of Rotating Blades

**DOI:** 10.3390/s24144480

**Published:** 2024-07-11

**Authors:** Angelos Filippatos, Simon Schwab, Tino Wollmann, Maik Gude

**Affiliations:** 1 Machine Design Laboratory, Department of Mechanical Engineering & Aeronautics, University of Patras, 26504 Patras, Greece; 2 Institute of Lightweight Engineering and Polymer Technology, Technische Universität Dresden, 01307 Dresden, Germany

**Keywords:** icing measurement, airfoils, volume reconstruction, ice volume, alpha shape

## Abstract

With the advancing energy transition, icing is a growing problem in the wind turbine sector. The development of systems to detect and mitigate icing makes it necessary to understand its basic behavior and characteristics. This paper proposes a method for the continuous and full-field measurement of the icing process of rotating blades, using a single line laser profile scanner. Inside of a climate chamber, a rotor is driven by a motor, while a system of nozzles provides a fine water dust, which leads to ice accumulating on simple NACA blades, which in turn is measured by a triangulation laser. The measurement data are cleared from outliers and presented as a surface in 3D space. An alpha shape is used to reconstruct and extract the volume of the ice between a reference and a measurement surface, using the corresponding Matlab function. Appropriate input parameters for the function and offsetting of the reference surface to improve the results are compared and discussed. The resulting system is able to detect small changes in the ice layer thickness in the sub-millimeter range.

## 1. Introduction

Icing is a common problem encountered in many fields of engineering. Wind turbine applications are one of them and with a globally expanding use of wind energy, its importance will rise over the next years [1]. Their high circumferential velocities make wind turbine blades prone to icing well before the rest of the structure [2]. The consequences of this are manifold and range from the shortened life spans of shafts or bearings, over financial losses, due to reduced power output from lower aerodynamic efficiency, up to high-risk safety hazards from falling or thrown chunks of ice [1,3,4,5,6]. The topic of icing is subject to standards like the ISO 12494 [7], that covers the general behavior and properties of ice, and the DNV-GL RO-0175, which is specifically addressing wind turbines [8].

The first step to develop systems that can detect icing or mitigate its effects is lab-scale research that allows one to study the effects of the ice itself or the effectiveness of systems for its detection or inhibition in a controlled environment. To correlate the effects to the icing, detailed information about its nature and distribution is necessary. Much work has been performed to study the ice accretion on airfoil structures, both rotating and stationary, on full-scale as well as small-scale models. However, so far, the main focus lies on the cross-sectional shapes of the leading edge ice. In contrast to in situ damage [9] and vibration [10], the analysis of rotors as well as the continuous and full-field monitoring of ice accumulation along the whole surface of the blade during an icing event has not received much attention so far, to the best of the authors’ knowledge.

Drapalijk et al. studied the ice accretion on small wind turbines in operation, with a blade length of up to 4 m, using cameras at different angles and approximating the ice as a tetrahedral volume, while using the known blade length as the calibration reference [11]. Experiments with a stationary blade section in an icing tunnel were carried out by Jin et al. [12], Hudecz et al. [13], and Hochart et al. [14]. While the advancing ice growth was recorded by Jin et al., using several cameras, the final results again focused on the cross-sectional leading edge structure of the ice. Han et al. performed ice accretion experiments on the small-scale rotating wind turbine blades to validate the prediction models for rime icing at the leading edge, thus focusing on the cross-sectional shape of the ice [15]. The guideline RP-0175 on the icing of wind turbines of the certification society DNV GL suggests a formula for ice mass distribution along a wind turbine blade for load modeling purposes, but again, the ice is vaguely assumed to be concentrated at the leading edge or the leading 30% of the blade surface [8].

Gantasala et al. investigated the effects of ice mass distributed along the blade of a small 1 kW wind turbine and trained a neural network to estimate the ice mass in three different zones, using the natural frequencies as an input, but the ice was placed manually on the blade [16]. A system that combines a natural ice accumulation with the possibility of measuring its distribution could be able to improve the validity of the results from such measurements.

Filippatos et al. proposed a test rig to measure the structural dynamic behavior of a rotating disc-rotor under icing conditions [17]. The experiments were conducted at a blade icing (BLICE) test rig, especially designed for this purpose. Water, sprayed from a nozzle system, leads to ice accumulating on the rotor. A laser scanner measured the ice on a radial line across the rotor. From the comparison of an ice free baseline and an iced measurement, the ice thickness and distribution on the rotor had been derived with high precision. For the work presented in this paper, the same BLICE test rig was used, but with a new designed bladed rotor, both in order to receive a 3D representation of the (iced) rotor blades and to compute the volume of the ice layers on the rotor blades.

The main challenges in data processing and analysis are the transition to a non-homogeneous rotational symmetry and icing behavior of the rotor, which means that the ice growth can no longer be reduced to an averaged 2D representation of its cross-section. Instead, it requires a three-dimensional representation of the surface data, and the reconstruction of a volume that envelops the point data measured by the laser scanner. The latter can be achieved using the mathematical tool of an “alpha shape”. While not originally designed for the purpose of surface or volume reconstruction, it has already been used for this in a variety of applications [18,19,20,21,22]. As a result, a novel method for 3D full-field in situ measurements of ice accreation and the associated data processing algorithms are demonstrated within this paper. The main novelty of this study is that it showcases the in situ icing process of rotating blades, using a single line laser profile scanner. The 3D representation of the (iced) rotor blades and the volume computation is performed in situ and enables the online monitoring of the process, thus enabling the monitoring engineer to possibly undertake the neccessary steps to avoid overloading the blades due to the increasing mass and hinder any structural damage. The general concept is explained in more detail in Section 2.5.

## 2. Materials and Methods

In the following, the design of the specimens and the components of the test rig used in the research for this paper are described. The main focus lies on the parts that are relevant for the induction of a natural icing process of the rotating specimens and the measurement of the ice layer.

### 2.1. Specimens

**Figure 1:**
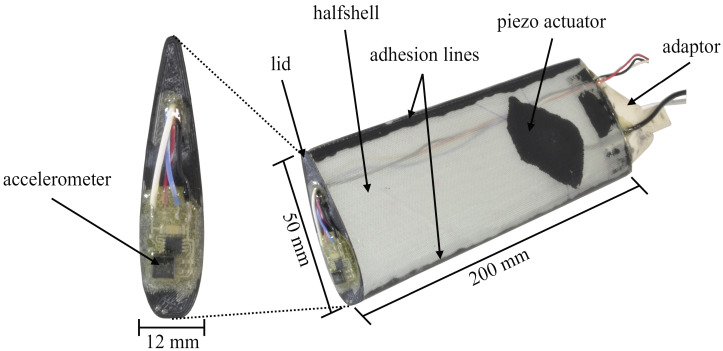
Components and dimensions of the rotor blade specimens.

The specimens, named Blade I and Blade II, are designed to follow a representative geometry of wind turbine blades and are manufactured in a vacuum-assisted resin infusion process. They consist of two separate shells that are glued together using the structural adhesive DP 490. A NACA 0018 resembles the relative thickness of the lower- to middle-part of the wind turbine blades. The dimensions of the specimens are defined by the space available in the test rig. To give them a slim shape at the given length of 200 mm, an airfoil depth of 50 mm was chosen. The thickness was also considered in this decision because the absolute thickness decreases with smaller depths but a certain amount of space was required to mount actuators and sensors inside the blades. The shells of the specimens consist of a four-layered stack of unidirectional glass fibers with angles in 0 and ±45° direction that are infused with the wind turbine certified epoxy resin hardener mixture RIMR 135, RIMR 137. At the root of the specimen, a 3D-printed Greentec Pro (Extrudr) adaptor is glued to attach them to the flange in the test rig. The tip is sealed by an also 3D-printed lid that prevents water intrusion into the blades and carries an acceleration sensor for vibration measurements. The sensor equipment as well as the piezoelectric macro-fiber composite actuator on the specimens are not relevant for the topic of this paper and are therefore not further explained. Figure 1 shows all components and the dimensions of the specimen.

### 2.2. Test Rig Set-Up

**Figure 2:**
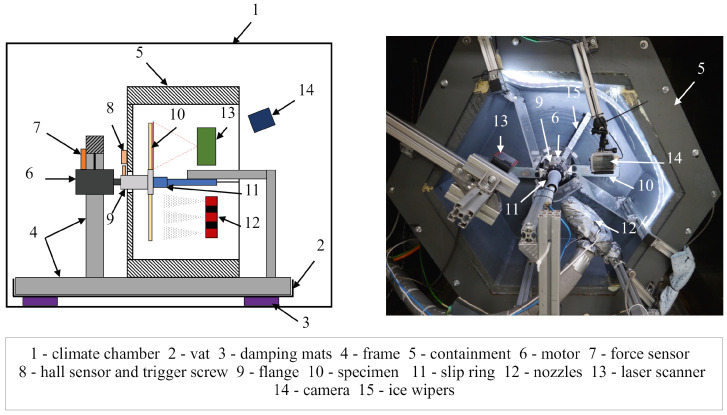
Sketch (**left**) and front view (**right**) of the test rig.

The complete set-up of the test rig, Figure 2, in detail is presented in [17]. Here, the focus lies on the relevant parts for the icing and laser profile scanning system.

The test rig is situated inside a climate chamber (1) to have control over the temperature conditions. Specimens can be mounted on the flange of a motor (9) and rotated at various velocities by a Kollmorgen AKM52L servomotor (6). Its speed can be set by an analog voltage signal that is fed to the corresponding Kollmorgen S712 frequency inverter that controls the motor.

**Figure 3:**
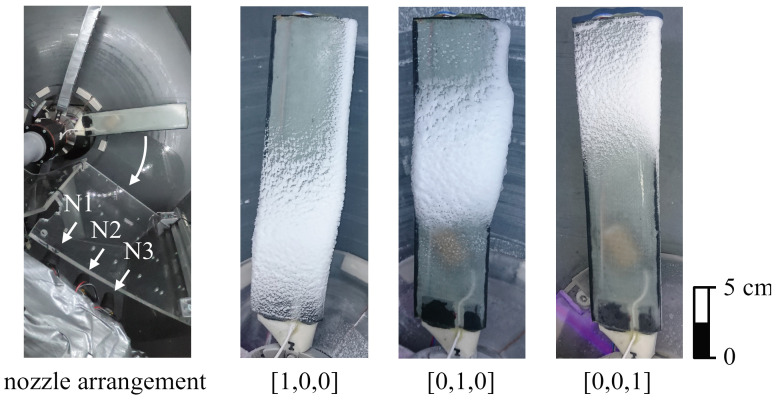
Nozzle arrangement and main icing area of each nozzle.

A system of three nozzles (12) perpendicular to the rotation plane allows the icing of the specimens at different radial positions. The water spraying process was performed continually, even during the measurements, to prevent the nozzles from freezing. The mass flow through the nozzles was manually controlled by adjusting their spray heads to values ranging from 3.5 to 4.5 $\frac{g}{\min}$. The value was increased from the inner to the outer nozzle to counter the higher circumferential velocity with the growing radius that leads to less ice accumulated for the same mass flow of water. The nozzles were operated at the lower end of their mass flow spectrum to create a very fine mist of water, which made them susceptible to the stress of the high-temperature gradients and led to frequent changes in the spraying properties. The nozzle setup is expressed by a binary vector whose values from left to right correspond to the nozzles from the inside to the outside and with a value of 1 meaning they are active. Figure 3 shows the resulting icing for each nozzle operating separately. The main icing areas are clearly visible.

The laser scanner (13), a Keyence LJ-V7300, provides information about the distance of an object that is crossing its measurement line by triangulation. It was developed for industrial high-speed scanning applications like orientation detection, quality assessment, or 3D-inspections. The device is oriented perpendicular to the rotation plane, but small deviations are negligible, because the calculation of the icing volume is based on the differences between the profiles of two measurements, therefore the absolute values do not matter. However, the alignment of the laser scanning line with the center of the rotation is much more important to achieve the correct results. The scanner is triggered to start and stop by a hall sensor (8) that detects a change in the magnetic field induced by a screw in the flange when passing by. The optical nature of the measurement method makes it best suited for opaque structures. There exist different types of ice that evolve under specific conditions for parameters like drop size, temperature, and relative velocity on impact [7]. With its glossy form, clear ice, which occurs at temperatures close to 0 °C, can lead to the high refraction of the laser beam and cause trouble in its detection.

An important point to consider with this and similar measurement techniques is the decreasing circumferential resolution $R_{u}$ with increasing radius $\left(R_{u}\propto \frac{1}{r}\right)$. This is directly linked to the angular resolution $R_{a}$ that can be controlled with the number of single-line measurements that are performed within one full rotation. The angular resolution therefore should be determined by the required resolution at the outermost part of the rotor:

(1) $$R_{u}=\frac{R_{a}}{2\pi r}.$$

### 2.3. Measurements and Data Organization

**Figure 4:**
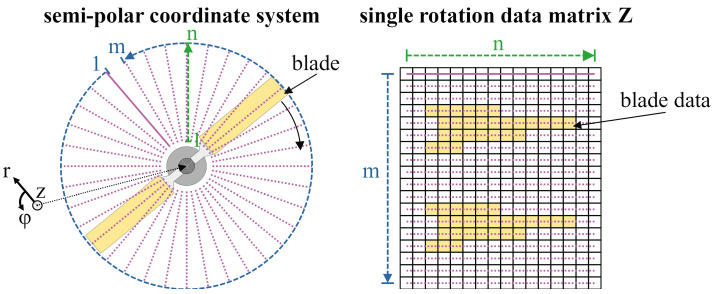
Schematic visualization of the composition of the measurement data from the laser scanner.

The laser scanner distance data are collected rotation wise in the form of the distance between the surface of the measured object and the scanner sensor. Each rotation is stored as a m × n matrix Z, where each row contains one scanned line while the columns represent the radial position as evenly spaced points along the laser line, as depicted in Figure 4. This means the data are originally stored in a semi-polar coordinate system. Over the course of a measurement, every second rotation is recorded. Depending on the measurement duration T and the rotational velocity $\omega_{\mathrm{rotor}}$, this leads to a maximum number of $k_{\max}$ single rotation arrays:

(2) $$k_{\max}=\frac{\omega_{\mathrm{rotor}}\times T}{2}.$$

These are stored in a txt-file format and later imported into Matlab R2023a, where they are concatenated to a three-dimensional array for further processing. Some arrays were omitted during the import, when they were found unsuitable, so the actual number of used arrays $k\leq k_{\max}$ can vary for each measurement.

### 2.4. Preprocessing and Presentation of the Ice Distribution

**Figure 5:**
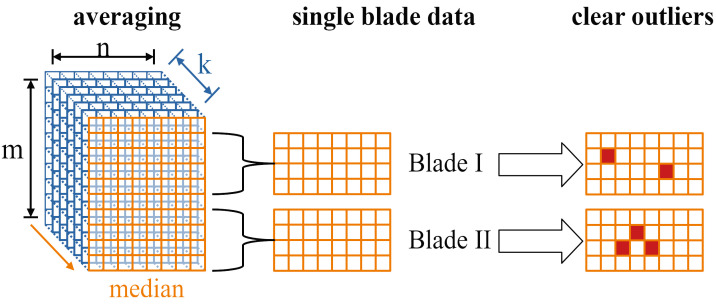
Schematic diagram of the most important preprocessing steps for the laser data.

All processing code was written in Matlab for an easy adaption of the algorithm and due to the availability of several useful functions for this project. An overview of the main steps of the preprocessing is given in Figure 5.

First, the values where the laser is measuring into the void are replaced with ‘NaN’ to segregate them clearly from the actual data. Furthermore, auxiliary elements of the rotor are removed in this step. After that, each single rotation array is scanned for big coherent data blocks, utilizing the function “bwconncomp” from the Matlab Image Processing Toolbox. This step is necessary to detect and remove datasets that contain more than one rotation, when the laser scanner does not catch the signal from the trigger to stop the measurement. Then, the remaining arrays of the current measurement are concatenated into the third dimension where each page stores the data for one full rotation. The number of valid entries for each point during the measurement is counted along the third dimension of the array to remove random noise in the form of small bits of data that are disconnected from the blades. Furthermore, measurements that are heavily shifted against the majority of the data are detected and omitted, by evaluating the overlap of the locations of the data entries in the arrays of the measurements. Then, the average blade profile is determined, using the median along the third dimension of the remaining data array to reduce the influence of outliers.

**Figure 6:**
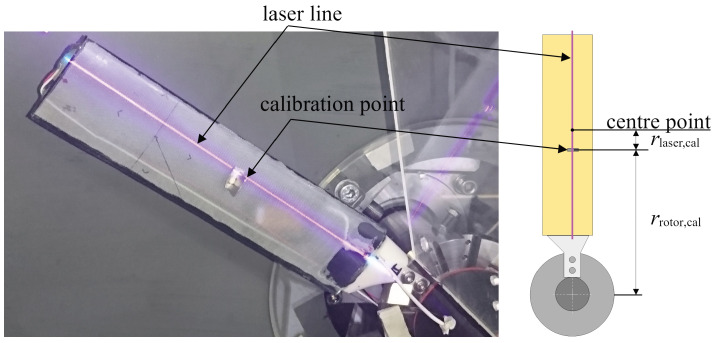
Sketch of the calibration parameters for the laser scanner.

The laser scanner inherently delivers the data in a semi-polar coordinate system, where the information about the radius and angle are contained in the indices of the column and the row of each entry. The radial resolution was 0.3 mm with values of the raw data running from −120 mm to 120 mm. The angular resolution is given by the number of m lines that are obtained within each rotation. A transformation into a Cartesian coordinate system is necessary for further processing and makes the handling and displaying of the data much easier. Therefore, two additional matrices X and Y of the same dimension as Z are needed to provide the x and y coordinates of the measurement points. In this step, the relative positioning of the laser and the rotor blades must be considered with a calibration value that is determined by mounting a small plate upright on a rotor blade, which creates a sharp peak in the laser scanner data, as shown in Figure 6. The difference between the measured peak and its real distance from the center of rotation yields the calibration value C. The entries of the matrices X and Y are calculated with the following equations, in which μ represents the row index, m represents the total number of rows, and ν represents the column index. The assignment of the sine and cosine operations to the matrices can be chosen arbitrarily, and this only affects the orientation of the resulting coordinate system.

(3) $$X_{\mu \nu }=\sin\left(\frac{2\pi \mu }{m}\right)\left(r_{\mathrm{laser},\nu }+C\right)$$

(4) $$Y_{\mu \nu }=\cos\left(\frac{2\pi \mu }{m}\right)\left(r_{\mathrm{laser},\nu }+C\right)$$

(5) $$C=r_{\mathrm{rotor},\mathrm{cal}}-r_{\mathrm{laser},\mathrm{cal}}$$

After the calculation of the x and y coordinates, the single-blade profile data can be extracted from the matrix without losing information. To achieve a standardized orientation for later calculations, the single-blade data are shifted to the origin of the coordinate system and its root edge is aligned with the y axis. This compensates for possible deviations in the position of the blades due to slight variations in the trigger of the laser measurement.

**Figure 7:**
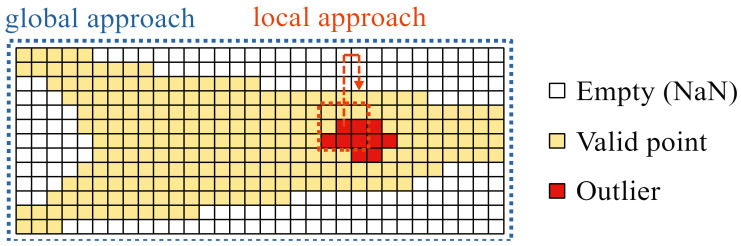
Schematic visualization of the measurement data array of one blade and different approaches for outlier detection using all the data at once (global) or small subsets (local). Outliers located close to each other, as depicted in red, can be challenging to detect with local methods.

Now the measurement data must be cleared from the remaining measurement errors because outliers in the point cloud can heavily influence the later computation of the volume, particularly for thin ice layers with a small overall volume. A multilayered process of outlier detection and removal is deployed, which combines global and local approaches, as depicted in Figure 7, using their individual advantages to compensate for the disadvantages of each other. The local approach evaluates the value of a point against its vicinity, which takes local effects into consideration, e.g., the given shape of the blades or ice accumulation. However, it is not useful when there are many outliers in a small area, because they “hide” each other when their values are of the same magnitude. A global approach is less sensitive to such effects, but can be too coarse to reliably detect all outliers and, in this specific case, is biased towards the blades root because the radially decreasing resolution leads to less data points at the blade tip.

**Figure 8:**
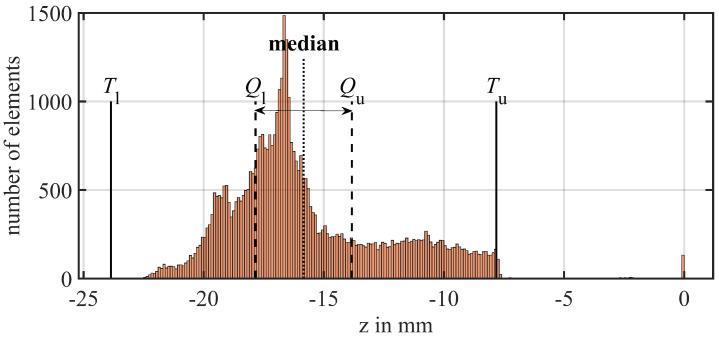
Exemplary histogram of an iced blade with the nozzle configuration [0,1,0] and thresholds from the quartile outlier detection method.

As a global technique, the Matlab function “isoutlier” is used to filter for the most obvious overall outliers. In this case, the quartile method is best suited to define incorrect values because the data are not normally distributed, which can be seen in the histogram for an iced blade in Figure 8. It defines upper and lower thresholds $T_{u}$ and $T_{l}$ for the data based on the interquartile range, which is the distance between the median $Q_{l}$ of the lower half of the sorted samples to the median $Q_{u}$ of the upper half of the samples:

(6) $$T_{u}/l=Q_{u}/l\pm 1.5\cdot \left(Q_{u}-Q_{l}\right)$$

**Figure 9:**
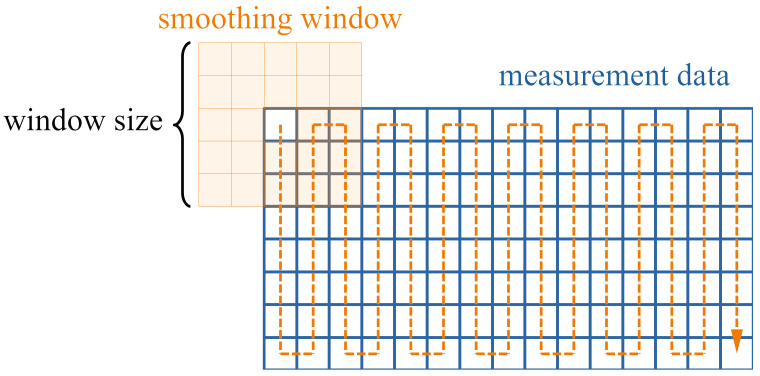
Smoothing of the data using a two-dimensional sliding window.

The local approach compares each data point to its surroundings, and was realized as a custom written function (The function was named “SmoothArea” and is available in the Matlab file exchange: [23]). For each point in the input array, the average is calculated within a specified window around the current point, which slides over the array and adapts to its the borders, as depicted in Figure 9. The absolute differences between the original and the averaged points are evaluated, using different thresholds, that were estimated based on the maximum slopes of the ice-free blade geometry, the used window size, and some inspection of the data.

In a first step, the global approach is used to filter for the most obvious outliers and exclude them from the following local evaluations. To reduce the number of false detection, as discussed earlier, the result is double checked with the local technique but using a big window size (21 × 21) and a high threshold of $T_{1}=2\;\mathrm{mm}$. All data points below the threshold are removed from the outlier pool again. The second step solely consists of the local approach, but with a smaller window size of 11 × 11. Outliers are now defined as points with a higher absolute difference to their corresponding average of $T_{2}=2\;\mathrm{mm}$. Last, the window is reduced to the immediate vicinity of the points (3 × 3) and the threshold is lowered to $T_{3}=0.5\;\mathrm{mm}$. Found outliers are omitted in the averaging process; therefore, the average can change each time the method is applied, and thus, the process is looped until no new outliers are found. Small bits of data (less than 10 samples) that become disconnected from the main block during the process are also labeled as outliers, because they lack trusted points around them to compare them with. When the detection process is finished, the outliers are replaced with linearly interpolated values. The Matlab function “fillmissing” is applied row and column wise to consider the properties of the specimens in the longitudinal and transversal directions. End values are not extrapolated because this can quickly lead to escalating distortions in the data when there are several missing values in a row. To give additional weight to the direct surroundings, a smoothed dataset with a window size of 3 × 3 is used as well. The filling process is looped two times because the independent use of the row and column wise filling can lead to the closing of gaps that were treated as end values and therefore not replaced the first time.

### 2.5. Ice Volume Reconstruction Using MATLAB Alpha Shape Function

**Figure 10:**
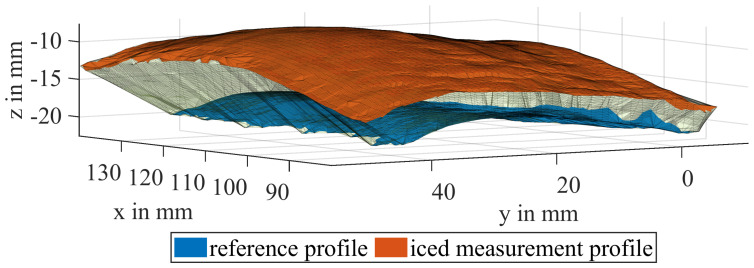
Partial volume of the iced mid-part of a blade, enveloped by the reference (blue) and a measurement profile (red).

**Figure 11:**
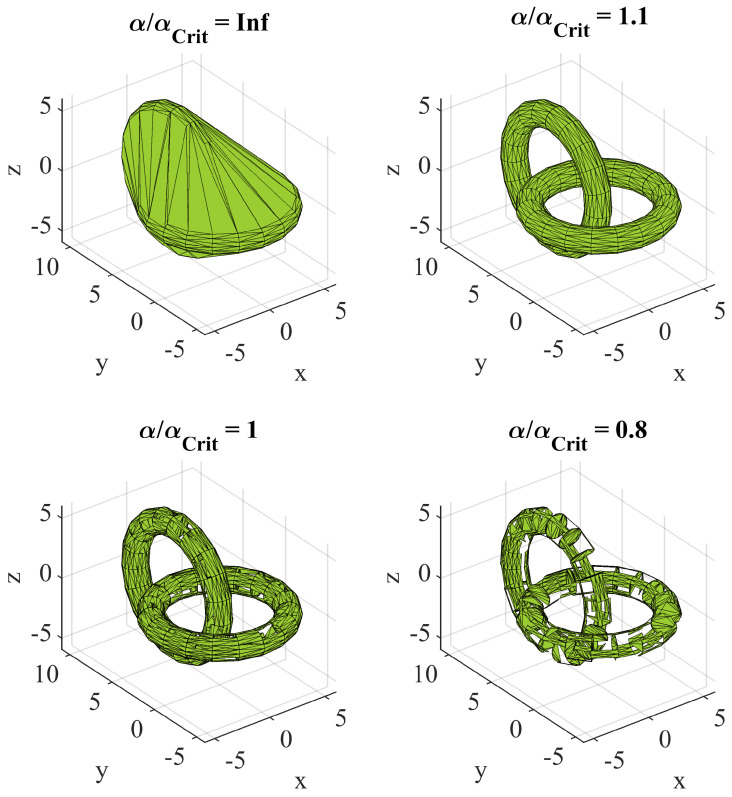
Effect of the α-radius on the resulting shape exemplary shown for two linked tori (based on [25]). An alpha that approaches infinity forms the convex hull of the point cloud.

The calculation of the ice volume requires the construction of a closed shape around a cloud of measured data points in 3D space, as depicted in Figure 10 for the iced middle part of a blade. The thickness of the ice layer varies across the blade, up to a maximum value of approximately 10 mm. The volume of interest is encapsulated between the surface area of the reference profile (blue) and the surface area of the iced profile (red), as shown in Figure 10. To extract the volume information from the point clouds, an alpha shape is created, which is a more general concept of the convex hull of a set of points P. Using the parameter α = [0,inf], a polytope is generated, whose facet size is dependent on the inverse of α. It can be imagined as the radius of an “eraser” that removes the connections of the polytope where it can fit without enclosing a point included at the set of points P [24]. An alpha shape creates a bounding area or volume that envelops a set of 2D or 3D points [19]. The alpha shape is also explained using the example at Figure 11 for better visualization purposes, to highlight the main idea that is a set of piece-wise linear simple curves in the Euclidean plane associated with the shape of a finite set of points. Another intuitive approach is the tightness of the fitting boundary that envelops the point cloud. The correspondent alpha shape to α = inf is the convex hull of P. With a decreasing α, the tightness of the fit of the enclosing shape is increased, until it ultimately only contains the points of P at α = 0.

Between these two extrema, there exist many different alpha shapes, as exemplarily illustrated in Figure 11. To use this mathematical construct as a tool for surface recreation requires the careful selection of the alpha parameter and process the data. The main steps are described in this chapter as well as the used configurations for this special case, but they can be adapted to fit different kinds of applications.

**Figure 12:**
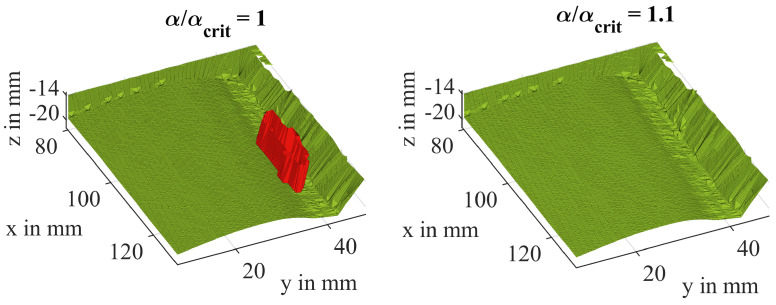
Boundary facets that cross the inside of the volume (**left**) can be suppressed by choosing the α value carefully (**right**).

Matlab provides an in-built function to compute an alpha shape object from the point cloud data. If no specified radius α is given to the function, it will determine it as the minimum radius $\alpha_{\mathrm{crit}}$ that is necessary to envelop all points. However, this does not necessarily guarantee that the resulting shape forms a closed volume around all points and is free from holes or interior surfaces. Depending on the sampling density, a value slightly higher than $\alpha_{\mathrm{crit}}$ should be chosen. This can also help suppress the creation of boundary facets that cut through the volume. In the case of irregularly distributed data points, α must be carefully selected to suppress holes while keeping as many small details in the resulting shape as possible (see Figure 12).

Due to the complex shape of the ice and the blade geometry in general, several steps are necessary to receive good results for the icing volume. The general approach consists of these main steps that are described in detail hereinafter:

1. Define the reference profile (first measurement of the series) and offset it, concatenate the reference with another measurement profile and determine $\alpha_{\mathrm{crit}}$;
2. Reconstruct edge icing by connecting the borders of the reference and the measurement surfaces with auxiliary points;
3. Create the alpha shape for the concatenation of reference, measurement and auxiliary points using α slightly bigger than $\alpha_{\mathrm{crit}}$ and extract the volume.

#### 2.5.1. Definition of a Reference Surface and Determination of $\alpha_{\mathrm{crit}}$

The reference surface is defined as the first profile of each measurement series. To ensure the proper functioning of the alpha shape algorithm for small differences between the reference surface and a measurement surface, we offset the reference data slightly by a value δ in the opposite direction of the icing. This leads to a better distinction between the reference profile and the surfaces of small layers of ice or ice-free parts of the blades and thus prevents the facets of the alpha shape to span over the concave leading edge of the blades. The offset should be chosen as low as possible but it should be higher than deviations in the measurement of the laser scanner, e.g., due to the slight out-of-plane motions of the rotor. The resulting volume offset is calculated from the alpha shape around the reference data with and without the offset and must be subtracted from all other measurements to compensate for the offset. For bigger layers of ice that exceed the original blade surface, an additional error can be expected due to a slight change in the angle of the lines that connect the borders of both surfaces, but this is assumed to be negligible compared to the impact of bad connections in the alpha shape and other uncertainties like measurement noise.

Additionally, the offset volume can be used to validate the algorithm that is used to construct the alpha shape. The linear shift leads to a volume $V_{\mathrm{off}}$ that can be compared to the analytical solution from the surface area *A* and the offset distance $dist_{\mathrm{off}}$:

(7) $$V_{\mathrm{off}}=A\cdot \delta .$$

**Figure 13:**
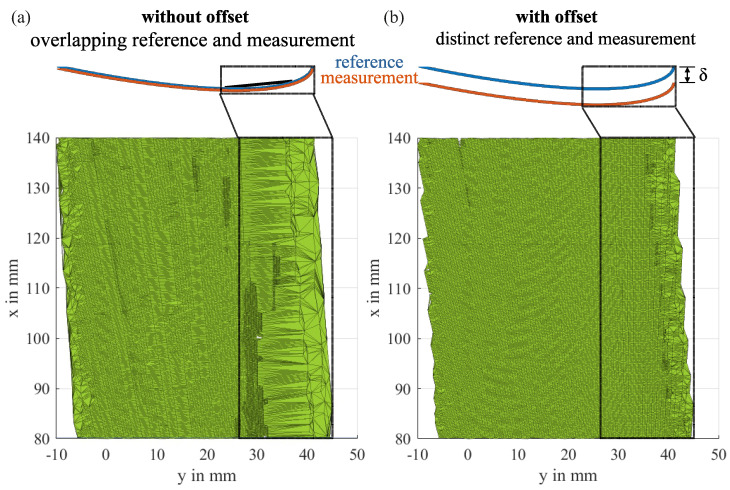
Bottom view of the rotor blades mid-part alpha shape, illustrating the effect of an offset reference profile: The facets on the concave leading edge, as well as the edges themselves, are much more detailed, when applying an appropriate offset (**b**).

Figure 13 shows the effect of the offset by comparing different ways to create the alpha shape for a section in the middle of the specimens. Should an ice-free measurement be chosen for the demonstration, the correct result for the ice volume would therefore be zero. All values for α were automatically determined by Matlab. For a better visualization of the effects, the pictures show the bottom-side of the resulting volume, the leading edge is on the right side. The left picture shows the resulting shape from the measurement data and the unchanged reference. The resulting shape fills a big portion of the concave leading edge which leads to an overestimation of the volume. To the right, the shape of the measurement with an offset reference is portrayed. The facets in the leading edge are much finer than before. This is due to a smaller α, which results in a better approximation of the measured volume. However, just reducing α for the case without an offset to obtain smaller facets can lead to a fragmented shape that does not envelop all data points.

The Matlab function “alphaShape” needs the point coordinates as an input. They can be presented as a matrix with three columns, in which each row contains the coordinate values of a point in the order x, y, and z. The reference and measurement data are concatenated and handed over to the function. Due to the fact that no α is defined, Matlab will automatically set it to $\alpha_{\mathrm{crit}}$. The value is derived from the alpha spectrum, which lists alphas that produce distinct shapes for the given data, where there is a notable change in the resulting shape. It can then be extracted from the properties of the resulting alpha shape object.

#### 2.5.2. Recreation of Leading and Trailing Edge Icing

**Figure 14:**
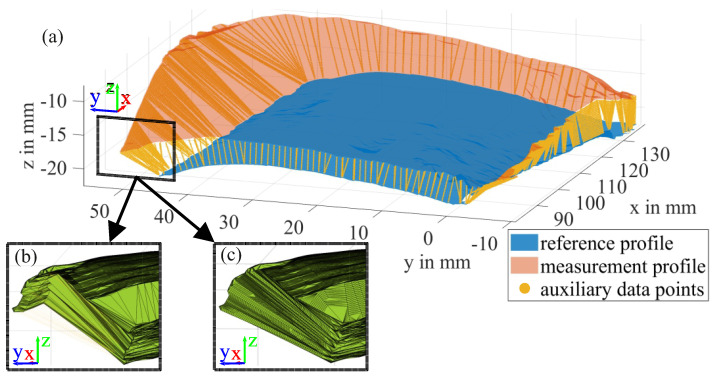
The iced measurement profile and the reference profile were connected with straight lines of auxiliary points along their borders (**a**) to avoid the creation of overhanging structures (**b**). The resulting shape is a better reconstruction of the leading edge icing (**c**).

With the progressive icing of the blades, the shape of the surface is changing. Especially the leading edge experiences much more icing due to its “cutting” motion through the mist. If the formed ice exceeds the reference surface, this can lead to overhangs, as depicted in Figure 14, which in turn can implicate significant underestimations of the actual volume. This problem arises from the demand for a small alpha-radius to obtain a detailed recreation of the surface structure. A straightforward solution to this is to fill the area between the two surfaces with auxiliary data points that prevent certain connections from being capped, and generating the alpha shape as desired while using a small alpha-radius. The auxiliary points in combination with a small alpha-radius would enable the recreation of the volume with a smooth transition between neighboring points. To illustrate the approach, straight lines were constructed between the border points of both surfaces. For each point in the dataset that has more border samples, the closest neighbor in the other set is determined and the connection line filled with equidistant points with a given target density of four points per millimeter, which is slightly smaller than the radial resolution of the measurement system.

#### 2.5.3. Create the Alpha Shape and Extract the Volume

As mentioned earlier, a set of points has a whole family of corresponding alpha shape objects to choose from, depending on α. The final alpha shape is created from the concatenation of the reference, measurement, and auxiliary points, with $\alpha =1.1\cdot \alpha_{\mathrm{crit}}$. It should be noted that the used factor was chosen arbitrarily based on the results of different examples and might differ for other experimental set-ups and data structures. A strict definition for α might lead to good results when changes in the spatial arrangement and differences in the density of the measured points are within a small range, which is not given for the present experimental design. $\alpha_{\mathrm{crit}}$ also varies strongly with the amount of ice on the rotor blades.

## 3. Results

### 3.1. Experimental Design

Two measurement campaigns were carried out at temperatures of −10 and −20 °C, each consisting of seven measurement series, one for each possible nozzle configuration. All series were aimed to contain about 40 measurements. The measurements were conducted every three minutes and lasted 40 s each. For each measurement series before the start of the icing process, three measurements were conducted to provide information about the initial conditions and the measurement deviations of the laser scanner. In all cases, the blades were rotated at a speed of 100 rpm, which translates into a tip velocity of 2.8 $\frac{m}{s}$.

**Figure 15:**
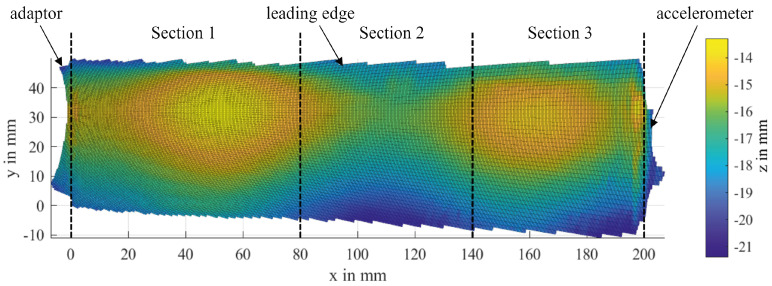
Sections for the volume calculation are defined according to the main icing areas (Series -20C_101).

To receive a high spatial resolution of the iced blades, the number of measured lines in each single rotation was set to 1080. The collected data were processed as described in Section 2.4 and Section 2.5. Three sections along the actual blade surface are defined with regard to the main icing area of each nozzle and their volumes are computed separately, as displayed in Figure 15. This has the positive side effect of lowering the overall difference in sampling density within each area, which leads to better adapted values for α.

The results for three different offsets, 0, −0.1 mm, and −0.5 mm, were computed and are further discussed in Section 3.3.

### 3.2. Results of the Preprocessing and Ice Distribution

**Figure 16:**
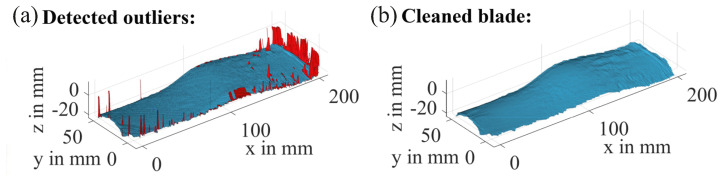
Found outliers during the preprocessing of the laser scanner data (**a**) and cleaned profile (**b**) for an iced blade with the nozzle configuration [0,1,0].

Exemplary results of the preprocessing for a measurement from the series -20C_010 are shown in Figure 16. All outliers were removed successfully while preserving most of the valid data. The resulting surface is a 3D representation of the ice distribution on the rotor blade with a high spatial resolution.

### 3.3. Comparison of Different Offsets for the Volume Calculation

**Figure 17:**
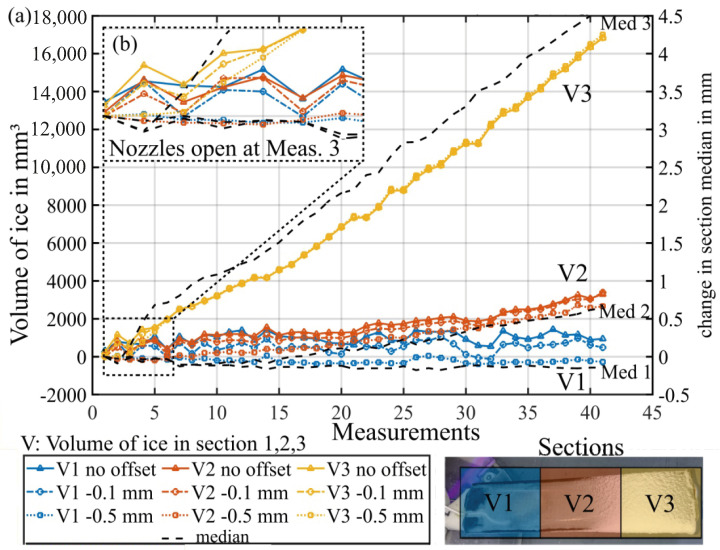
Comparison of different offsets for the reference surface for exemplary data from Blade II with the nozzle configuration [0,0,1].

Figure 17 shows the calculated ice volumes V of Blade II for the nozzle configuration [0,0,1] at a temperature of −20 °C for three different offsets for the reference data, as well as the trends of the median values of each blade section. The main scope of this comparison is to show how the results are affected from the selected offset, as this offset is required to calculate proper results close to the measurement noise, and especially at the beginning of the measurements, where the ice thickness is close to zero. For the cases where an offset was applied, the effect of the offset was compensated by subtracting the offset value from each measurement. These partially led to negative values, as shown in Figure 17b. The presented nozzle configuration was chosen for the reason that the innermost blade section can be expected to remain totally free from ice. As it can be seen in Figure 3, all blade sections further out from the active nozzle experience leading edge icing to some extent. Additionally, at a certain point, the ice may exceed the defined borders of the section and is partially counted to the neighboring section(s). The expected qualitative trends for the blade sections are as follows:

- No ice accumulation in Section V1, shown as V1, blue color in Figure 17a, between measurements 01 and 20, with a slightly increase from measurement 21 to 41.
- Slight ice accretion in Section V2, shown as V2, towards the end of the measurement series due to ice growth across the border to Section V3.
- Strong icing in Section V3 due to the active nozzle, directly spraying at the respective area.
- No icing during the first three measurements due to closed nozzles, as shown in Figure 17b.
- The deviations in the first two measurements can be considered noise. From measurements 3 to 41, there is a clear increase in the ice in Section V3.

The overall trends of the volumes match the expectations. As expected, the impact of the offset is small, when there is enough ice accretion on the blade, as it is the case for Section V3, because the additional ice layer exerts the effect and allows for a clear distinction between the measurement and the reference surface. Section V2 also experiences converging results, as the ice starts to grow over the border from Section V3. The remaining difference can be explained by the effect, that the ice does only partially cover the section (see transition zones in Figure 15). This leaves the ice-free part prone to flawed connections in the alpha shape at the concave leading edge, as discussed in Section 3.3.

The advantage of setting an offset can be seen for Section V1, where there is a significant decrease in noise due to the reconstruction with the alpha shape, as it lowers the resulting volumes by up to 1500 ${\mathrm{mm}}^{3}$ for an offset of −0.5 mm. This leads to remarkably smoother results, illustrating the need of the offset in low ice areas.

To finally determine which results are most trustworthy, they are qualitatively compared to the deviations of the median values of the corresponding sections. While the median is not a suitable parameter to determine the actual volume, it can give an overall hint on the icing trend, as it is shown by the correlation in Section V3. For Sections V1 and V2, negative median trends can be observed, which can be caused by tilting motions of the rotor out of its plane. In Section V1, it is advancing over the whole measurement series, which suggests that there might be some kind of a rotor displacement that is influenced by the growing ice. However, the change in the median stays within a small range of a fraction of a millimeter, and is quite small compared to the influence of the ice, but with a range of up to −0.18 mm, it is still big enough to compensate the applied offset in the case of −0.1 mm. This has visible implications on the resulting volumes, that are quite similar to the ones calculated without an offset and do not follow the same trend as the change in median, which again is attributed to bad connections in the alpha shape when the reference and measurement surface are not distinct. However, the offset of −0.5 mm is big enough to withstand the compensation, which allows more stable results for the volumes with a good correlation to the median change. This leads to the conclusion, that, for the measurements conducted in this paper, the offset of −0.5 mm leads to the results with the highest confidence, and is used for all the results presented as follows.

### 3.4. Icing of the Blades

In general, the icing only took place on the upper blade surface that was facing towards the nozzles and at the leading edge, while the lower blade side remained ice-free. To determine the type of ice on the specimens, the following parameters were used and compared to the characteristics in [7]:

- Ambient temperature;
- Visual appearance;
- Density.

Different types of ice were observed for both measurement campaigns. For a temperature of −20 °C, the probability of forming soft rime ice is very high. This is further substantiated by the white opaque appearance and the low density of 0.342 $\frac{g}{{\mathrm{cm}}^{3}}$ on average (see Table 1). At temperatures of −10 °C, both soft and hard rime ice are possible. In that case, the slightly transparent appearance and the much higher density indicate the latter one, but a mixture of both seems also possible. The calculated densities are plausible and match the expectations based on the temperatures. This is taken as an indicator for the correctness of the calculated volumes.

**Figure 18:**
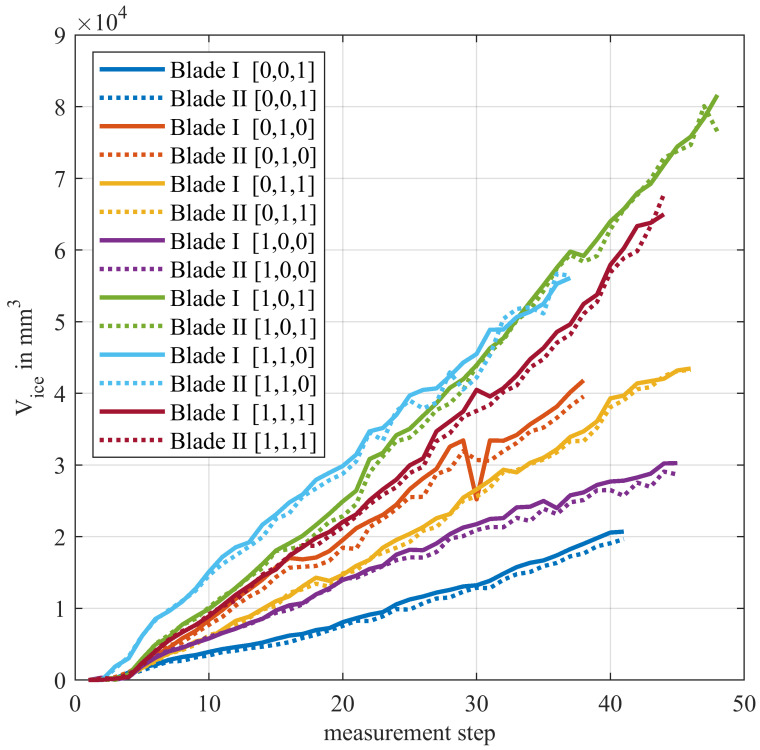
Total ice volumes of both blades for the measurement series at −20 °C.

In general, the ice accumulation happened evenly and almost linearly on both specimens. This can be seen in Figure 18, which shows the cumulative ice volume over all three parts of the blades, calculated with a reference offset of −0.5 mm. Negative volumes, as they were discussed in the chapter before, were set to zero, otherwise they would distort the results. The high level of consistency between both blades suggests a good quality of the underlying measurement data. The linear ice growth matches the results of a study regarding the general behavior of rime ice on structures by Makkonen et al. [26]. The sharp drop in the 30th measurement step of Blade I for -20C_010 is due to heavily damaged raw data.

As expected, a higher number of open nozzles tends to lead to a faster icing of the blades. However, another import factor, which can overcompensate the active number of nozzles, is the mass flow through the nozzles that were prone to frequent changes (see Section 2.2). Also, icing at the tip of the blades was much harder to achieve than at the root, despite a higher mass flow through the according nozzle and the higher circumferential speed. This indicates, that the cutting motion of the blade through the water mist plays a subordinate role for the icing process. The speed of the water droplets is mainly influenced by their exit velocity at the nozzles. This means that, for this set-up, the retention time of the blades in the water spray, which become lower from the root to the tip of the blades, is crucial for the ice accumulation.

The precision of the ice detection regarding the average layer thickness can be estimated from the blade surface area and the volume gradients between the measurement steps. Based on the present measurement results, it lies within the sub-millimeter range.

## 4. Discussion and Conclusions

The test rig and data processing procedures described in this paper were found to be capable of inducing natural ice growth on rotating blade specimens and measuring the consequential ice distributions and volumes. It is able to detect icing on the sub-millimeter scale. The presented approach for the volume calculation, based on Matlabs inbuilt “alphaShape” function, makes it easy to implement. The level of detail in the volume reconstruction might be improved using more sophisticated algorithms that adapt α based on the local sampling density as proposed in [20]. On the other hand, this might be more computationally expensive. The resulting volumes were found to be very plausible, but further work can be performed to exactly evaluate the precision of the volume calculation. Comparisons to numerical models for ice accretion would also be interesting, but to match the experimental and modeling results, the test rig might have to be expanded to be capable of measuring the size and speed of the water droplets.

Another field with room for improvement is the reconstruction of the leading edge icing. The presented measurement arrangement is not able to completely catch the ice structures there. This might be solved with another profile scanner that operates in the opposite direction as the first one and provides a backside view of the specimen. Alternatively, analytical formulas that approximate the leading edge ice shape could be used for a more realistic reconstruction [27]. However, the implementation might be challenging.

It seems possible to adapt the proposed system for other tasks as well, e.g., to detect the accumulation of dirt, abrasion, de-icing processes or other effects that change or deform the surface of a surveyed object. While the data generation and processing was performed offline in this paper, it would be possible to adapt it for continuous online surveillance, if necessary.

## Figures and Tables

**Table 1:** Overview of the conducted measurements (volumes calculated with an offset of −0.5 mm).

Series Name	Nozzle Config.	Temperature [°C]	Rotational Velocity [RPM]	Final Ice Mass [g]	Total Ice Volume [cm^3^]	Ice Density $\left[\frac{g}{{\mathrm{cm}}^{3}}\right]$
-20C_001	[0,0,1]	−20	100	13.5	49.762	0.271
-20C_010	[0,1,0]	−20	100	30.0	91.476	0.328
-20C_011	[0,1,1]	−20	100	31.0	96.840	0.320
-20C_100	[1,0,0]	−20	100	27.0	69.478	0.389
-20C_101	[1,0,1]	−20	100	63.0	168.123	0.375
-20C_110	[1,1,0]	−20	100	44.5	122.756	0.363
-20C_111	[1,1,1]	−20	100	50.0	142.904	0.350
-10C_001	[0,0,1]	−10	100	9.5	22.507	0.422
-10C_010	[0,1,0]	−10	100	25.5	42.280	0.603
-10C_011	[0,1,1]	−10	100	44.0	69.572	0.632
-10C_100	[1,0,0]	−10	100	41.5	58.779	0.706
-10C_101	[1,0,1]	−10	100	52.0	77.214	0.674
-10C_110	[1,1,0]	−10	100	36.0	53.844	0.669
-10C_111	[1,1,1]	−10	100	51.0	74.840	0.682

## Data Availability

Dataset available on request from the authors.
